# Women-specific HIV/AIDS services: identifying and defining the components of holistic service delivery for women living with HIV/AIDS

**DOI:** 10.7448/IAS.16.1.17433

**Published:** 2013-01-11

**Authors:** Allison J Carter, Sonya Bourgeois, Nadia O'Brien, Kira Abelsohn, Wangari Tharao, Saara Greene, Shari Margolese, Angela Kaida, Margarite Sanchez, Alexis K Palmer, Angela Cescon, Alexandra de Pokomandy, Mona R Loutfy

**Affiliations:** 1 Simon Fraser University, Faculty of Health Sciences, Burnaby, British Columbia, Canada; 2 British Columbia Centre for Excellence in HIV/AIDS, Vancouver, British Columbia, Canada; 3 Women's College Research Institute, Women's College Hospital, University of Toronto, Toronto, Ontario, Canada; 4 McGill University Health Centre, Montreal, Canada; 5 Women's Health in Women's Hands Community Health Centre, Toronto, Ontario, Canada; 6 McMaster University, Faculty of Social Work, Hamilton, Ontario, Canada; 7 ViVA, British Columbia, Canada; 8 All additional research team members are listed at the end of the article

**Keywords:** HIV, women, gender, women-specific services, women-centred care, HIV/AIDS programming, health services, CHIWOS

## Abstract

**Introduction:**

The increasing proportion of women living with HIV has evoked calls for tailored services that respond to women's specific needs. The objective of this investigation was to explore the concept of women-specific HIV/AIDS services to identify and define what key elements underlie this approach to care.

**Methods:**

A comprehensive review was conducted using online databases (CSA Social Service Abstracts, OvidSP, Proquest, Psycinfo, PubMed, CINAHL), augmented with a search for grey literature. In total, 84 articles were retrieved and 30 were included for a full review. Of these 30, 15 were specific to HIV/AIDS, 11 for mental health and addictions and four stemmed from other disciplines.

**Results and discussion:**

The review demonstrated the absence of a consensual definition of women-specific HIV/AIDS services in the literature. We distilled this concept into its defining features and 12 additional dimensions (1) creating an atmosphere of safety, respect and acceptance; (2) facilitating communication and interaction among peers; (3) involving women in the planning, delivery and evaluation of services; (4) providing self-determination opportunities; (5) providing tailored programming for women; (6) facilitating meaningful access to care through the provision of social and supportive services; (7) facilitating access to women-specific and culturally sensitive information; (8) considering family as the unit of intervention; (9) providing multidisciplinary integration and coordination of a comprehensive array of services; (10) meeting women “where they are”; (11) providing gender-, culture- and HIV-sensitive training to health and social care providers; and (12) conducting gendered HIV/AIDS research.

**Conclusions:**

This review highlights that the concept of women-specific HIV/AIDS services is a complex and multidimensional one that has been shaped by diverse theoretical perspectives. Further research is needed to better understand this emerging concept and ultimately assess the effectiveness of women-specific services on HIV-positive women's health outcomes.

## Introduction

**Figure 1:**
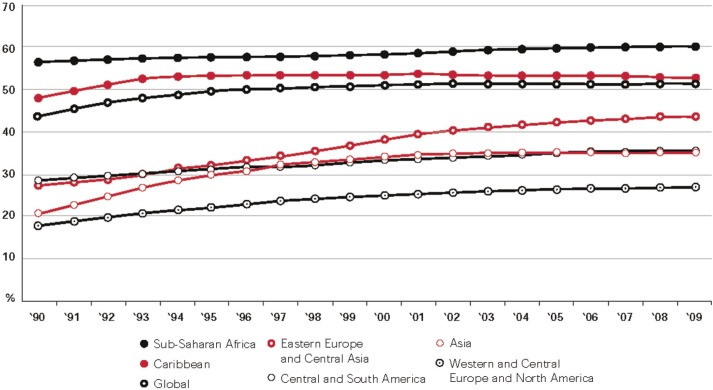
Proportion of people living with HIV (WLWH) who are women, 1990–2009. Reproduced with permission from UNAIDS [5].

The profile of the global HIV/AIDS epidemic has changed dramatically over the past three decades, from a disease that predominantly affected men to one that is affecting a growing number of women. Women now represent over 50% of the 33.3 million people living with HIV globally [1]. In regions of sub-Saharan Africa, women constitute a disproportionate 60% of HIV cases [1]. In Latin America and the Caribbean, the percentage is over 35 and 50%, respectively [1]. In Asia, the proportion of women living with HIV (WLWH) has grown even more rapidly. In China, for example, the male-to-female sex ratio among HIV-positive people has narrowed from 9:1 in the 1990s to 3:1 in 2007 [2,3]. In North America, men who have sex with men continue to account for the majority of people living with HIV, but the proportion of WLWH has steadily increased over the past decade. In Canada, 26% of newly diagnosed HIV infections in 2009 were among females aged 15 years and above, more than double the proportion observed in 1999 (12%) [4]. Figure 1 shows the increasing proportion of WLWH globally over time [5].

Differences in the biological and social realities of men and women are key drivers of the feminization of the HIV epidemic [6]. In the context of heterosexual vaginal intercourse, the efficiency of male-to-female HIV transmission is two times greater than female-to-male transmission, owing to a more receptive contact surface of the vagina, a higher concentration of HIV in semen compared to vaginal fluid and cervical ectopy [6,7]. Social factors can exacerbate this increased risk [8,9]. For instance, women who are economically disadvantaged [10–12] or who have experienced gender-based violence [13–15] are more likely to engage in unprotected sex, have multiple partners and resort to trading sex for money, drugs, food or housing. These women are also less likely to have the capacity to affirm one's self and to negotiate condom use, discuss fidelity with partners and leave risky relationships [10–15].

Access to and maintenance in treatment also varies by gender, both globally [16–19] and in Canada [20–22]. WLWH experience several barriers to care which are heavily shaped by gender, including stigma and discrimination (such as HIV-related stigma, sexism, racism and homo/transphobia) [23,24], violence [25], mental health and addiction issues [26], a lack of financial resources [27,28], lack of social support and feelings of isolation [26], inflexibilities in clinic hours [29–31], negative experiences with health care providers [32], a lack of services focusing on women [33], long travel distances to services from rural or remote areas [28,34,35] and competing responsibilities as mothers, partners, friends, homemakers, paid-workers and care-givers in which women prioritize the needs of others above their own [36,37].

Conflicting results have been published in terms of sex differences in outcomes after treatment initiation [38,39]. While some authors have reported improved virological suppression in males [40], others have showed advantages in females [41–44]. However, most evaluations have found no sex differences after adjustment for confounding variables [45–49]. Nevertheless, women are more likely to be non-adherent, have treatment interruptions and experience more adverse drug reactions [38,39]. Also, HIV infection increases the severity of menopause and menstrual disorders, osteoporosis, pelvic inflammatory disease and vulvo–vaginal and cervical diseases [38,50].

Women also have distinct reproductive health concerns, including contraception, fertility and pregnancy [38,50,51]. Provision of the full range of contraceptive options and access to safe abortion services are critical components of care to prevent unplanned pregnancies and improve women's overall health [52]. It is similarly critical to support women to safely achieve their future reproductive goals through pre-conception, pregnancy and post-partum services and support (including access to fertility treatment services as required), as an increasing number of HIV-positive women express the intention to have biological children [53]. In addition to reproductive concerns, sexual satisfaction, sexual functioning and sexual negotiation are increasingly important concerns to address as WLWH have been wrought with sexual stigma and a near absence of supportive services [54,55].

Women's experiences of HIV infection are unique and tailored services that respond to women's needs are critical for improving the overall health outcomes of WLWH [6]. In response, some regions have created women-specific HIV/AIDS programmes and services. For instance, in Canada, some of these include the Oak Tree Clinic, Positive Women's Network, the Maple Leaf Medical Clinic, Women's Health in Women's Hands Community Health Centre and the Centre for AIDS Services of Montreal Women; in the United States (US), the Johns Hopkins HIV Women's Health Program, the Women's Collective, SisterLove Inc. and US Positive Women's Network; and globally, Women Fighting AIDS in Kenya, Mama's Club in Uganda, Mujeres Positivas in Latin America and Women Organized to Respond to Life-threatening Diseases. Despite the emergence of this model of care, the concept of women-specific HIV/AIDS services remains largely undefined. These approaches are not well conceptualized and little is known about the key characteristics of women-specific HIV/AIDS services.

Accordingly, the objective of this investigation is to explore the concept of women-specific HIV/AIDS services to identify and define what key elements underlie this approach to care. This literature review was undertaken as part of the formative phase of a new community-based, prospective cohort study entitled the Canadian HIV Women's Sexual and Reproductive Health Cohort Study (study acronym: CHIWOS; study website: www.chiwos.ca). This study aims to enrol 1250 HIV-positive women from three Canadian provinces, including Ontario, Quebec and British Columbia, with plans to expand to Nova Scotia, Manitoba, Saskatchewan and Alberta. Understanding the concept of women-specific HIV/AIDS services is critical to our research, and more generally, to better addressing the unique needs of WLWH.

## Methods

### Theoretical framework

Our approach to understanding women-specific HIV/AIDS services emphasizes the relationship between many interacting social factors and women's health. While good medical care is vital, the underlying social and structural factors that undermine women's health must be addressed to have an opportunity for good health and wellbeing. Hence, the services that will be most effective in meeting the needs of WLWH will be those that reflect the intersectional social positions that women occupy in society. This understanding has resulted in our decision to ground our research in a Social Determinants of Women's Health (SDoWH) framework [56,57]. This gendered framework recognizes the high degree of variability between women, men and other gender groups, as well as between and within groups of women, based on intersections of identity and the lived social, economic and political realities, and the role that these intersections play on shaping individual health outcomes and experiences with health services [58,59]. In the context of HIV, this framework allowed us to recognize how multiple identity statuses and social factors are always at play in the lives of WLWH, and how this inter-sectionality affects their HIV/AIDS service needs and experiences in complex ways [60].

### Search methods

This review was written using scoping review methodology [61]. A comprehensive literature search was conducted using online databases, including CSA Social Service Abstracts, OvidSP, Proquest, Psycinfo, PubMed and CINAHL. The first search included the following key words: “women-specific”, “services for women”, “HIV-positive women”. After a review of the articles retrieved, our search strategy was expanded to include “female-specific”, “women-focused treatment”, “women-only services”, “programmes for women”, “gender responsiveness”, “women-centredness”, “women's needs” and “gender awareness”. The terms were used both alone and in all possible combinations. After this initial retrieval of articles, additional articles were reviewed from the reference lists of articles eligible for inclusion. An Internet search was also conducted to locate grey literature, such as reports about women-specific models of service delivery from across Canada and elsewhere.

### Inclusion criteria

All articles and reports identified were reviewed by two researchers (AJC, SB). These researchers were also responsible for discussing disagreements over eligibility until a consensus was reached. To be included for review, articles had to feature women-specific services as their central focus and make a contribution to the review aim of exploring the concept of women-specific HIV/AIDS services. Articles were restricted to English language publications with no limit set on the date or place of publication. Owing to limited literature on this topic as revealed in our preliminary search, we considered articles from various subject areas if they explored women-specific services in general and we discussed implications for the field of HIV/AIDS. While the rubric outlined by other disciplines may not perfectly translate to the context of HIV/AIDS, we believe this to be a reasonable approach since, from a SDoWH perspective, multiple issues and concerns besides HIV are always at play in the lives of WLWH. Thus, investigating a range of service models from several disciplines allowed us to better understand this concept for women in all of their diversity.

## Results and discussion

The initial literature search generated 84 peer-reviewed articles. Of these, 22 met the inclusion criteria and were included for full review. After augmenting the search further, five articles were retrieved from article reference lists and three reports were obtained through a general internet search, for a total of 30 articles included in this review. Articles were published between 1995 and 2010. Most originated from Canada and the United States. Fifteen were specific to HIV/AIDS, eleven to mental health and/or addictions and the remaining four stemmed from other subject areas. Table 1 outlines the source of each article by country of origin and discipline.

**Table 1:** List of identified articles exploring women-specific services by region and subject area (total number of articles identified=30)

	*n* (%)	References
Region		
United States	20 (67%)	[62–80]
Canada	6 (20%)	[81–86]
Others (United Kingdom, Australia)	4 (13%)	[87–90]
Subject area		
HIV	15 (50%)	[8,63,67,70–72,76–78,80,82,84–86,90]
Mental health and/or addictions	11 (37%)	[62,64–66,68,69,74,75,79,88,89]
Women's health in general	2 (7%)	[73,81]
Cardiovascular health	1 (3%)	[83]
Law and policy	1 (3%)	[87]

### Defining features of women-specific HIV/AIDS services

The review demonstrated the absence of a consistent, widely accepted definition for women-specific HIV/AIDS services. Within the literature, this approach to care was named and defined in multiple ways. In addition to “women-”, “sex-” or “gender-specific” services, other terms commonly used included “women-only”, “(tailored or specialized) programming, programmes, services or treatment for women”, “women-centred”, “women-focused”, “women-” or “gender-sensitive”, “single-gender” or “single-sex”, “women-” or “female-friendly”, “family-focused”, “family-friendly”, “same-gender” or “same-sex”, “women's health services”, “transformative”, “empowering”, “women-exclusive”, “gender-responsive”, “gender-appropriate” and gender-equitable” approaches. Table 2 shows the frequency with which these terms were used in the literature reviewed.

**Table 2:** Comparison of terms used to describe women-specific services

Term	*n* *	References
“women-”, “female-“, “sex-” or “gender-specific”	15	[62,63,65,68–70,72,73,76,77,82,85,87,89,91]
“women-only”	15	[62,64–66,68,69,74,78,79,81–83,85,88,89]
“(tailored or specialized) programming, programmes, services or treatment for women”	15	[62,64,66–71,74,75,77,84,87,89,91]
“women-centred”	9	[67,68,72,77,81,83,85,87,88]
“women-focused”	8	[62,64,67,68,73,74,78,83]
“women-” or “gender-sensitive”	8	[8,62,66,68,76,81,88,91]
“single-gender”, “single-sex”	6	[62,64–66,88,89]
“women-” or “female-friendly”	4	[66,86–88]
“family-focused”	4	[68,70,72,80]
“family-friendly”	3	[70,80,84]
“same-gender”, “same-sex”	2	[62,66]
“women's health services”	2	[73,91]
“transformative”	2	[8,76]
“empowering”	2	[8,76]
“women-exclusive”	1	[85]
“gender-responsive”	1	[62]
“gender-appropriate”	1	[77]
“gender-equitable”	1	[63]

* Many articles used more than one term to describe women-specific services. Hence, the accumulative numbers shown in this table (96) exceed the total articles reviewed (30).

A useful starting definition comes from Grella [62], who described women-specific services as those offered to women only or those in which there is a higher concentration of female clients. Examples of the former may include gynaecological, breast health or menopausal services, all of which are important issues for WLWH [51]. It may also include a range of other gender-neutral services, if offered in a women-only environment [62]. An example of the latter may be HIV-positive parent–baby groups that are attended by mostly women. Furthermore, while some women have expressed that having staff that are competent in and sensitive to HIV- and women-specific issues is more important than having female providers per se (Margarite Sanchez, Personal Communication, February 2012), others have emphasized that having one's identity reflected back at them is a crucial component to care for women [29,65]. Thus, the primary, defining and consistent feature of women-specific HIV/AIDS services is the gender of the clientele and/or staff. In addition, these services are either provided within larger single- or mixed-gender settings [62,65].

However, many authors [62,65,68,69,78,79,83,84] suggest that women-specific services “must do more than segregate clients and employ only female staff” [65]. Several studies have provided empirical support for this hypothesis. For example, in a retrospective, quasi-experimental cohort study of a drug treatment programme that segregated clients and staff but left the programme content unchanged, there were no significant differences in treatment outcomes between participants enrolled in single- or mixed-gender groups [65]. In contrast, Claus and colleagues [68] demonstrated that substance-using women treated in women-only settings with specialized programming for women (e.g. childcare services, education on women's health topics) had better outcomes after discharge compared to women treated in non-specialized, mixed-gender programmes.

Therefore, in addition to the provision of care and support in an all-female environment, many authors argue that, to be effective, women-specific services must also adopt approaches to care that are substantially different than the traditional care provided in mixed-gender settings [62,65,68,69,78,79,83,84]. This is based on recognizing that women are unique and therefore have health and social care needs that require specially designed programmes. It also is based on an understanding of the close connection between women's health and their whole lives [58,59,73,81,92]:

Women's health involves women's emotional, social, cultural, spiritual and physical well-being, and is determined by the social, political, and economic context of women's lives as well as by biology. This broad definition recognizes the validity of women's life experiences and women's own beliefs about and experiences of health. Every woman should be provided with the opportunity to achieve, sustain and maintain health, as defined by the woman herself, to her full potential. [92] (pp. 507–508)

Following from this, findings from the literature suggest that there are at least 12 additional components to women-specific HIV/AIDS services that are important for holistically addressing women's needs and promoting women's health. Drawing on the work done by the Vancouver/Richmond Health Board [81], which was informed by several women's health centres from across Canada and elsewhere, we distilled these elements into four different categories and now discuss each element to demonstrate how they may be applied in practice.

Importantly, these principles are presented with the understanding that some features of good HIV/AIDS service delivery may apply to women only and others may be universally relevant to both men and women. Hence, while we explore all the potential aspects of HIV/AIDS services for women, we appreciate that they are not necessarily exclusive to women-specific services and may be important in the care of men as well. In addition, we present these principles with the acknowledgement that they will evolve as additional research is conducted. It is also recognized that the degree to which these principles is achieved in practice may vary depending on the context, purpose and patients involved. Finally, although presented here as distinct items, it is important to recognize that many of the components overlap and are related to each other; women-specific HIV/AIDS services actually involve an integration of some or all of these items, which together impact the entirety of women's experience accessing care.

## Additional dimensions of women-specific HIV/AIDS services

### Strategies to successfully engage WLWH

#### Creating an atmosphere of safety, respect and acceptance

Some HIV-positive women have reported underutilizing health and supportive services because of negative experiences with providers where they have felt unsafe, unwelcome or discriminated against [29,30,32]. Other women, in the context of sex work, have reported avoiding care because of stigma, criminalization or fear of running into aggressors [29,93]. Thus, addressing the health of WLWH begins with safety, respect and acceptance [81,85,88]. This involves creating inclusive, welcoming and non-competitive women-only spaces where women feel comfortable sharing potentially sensitive and painful issues [64,66,68,83]. It also requires providers to recognize the different kinds of abuse experienced by WLWH [29,30] and to understand their subsequent coping strategies [81]. In such an approach, providers are encouraged to take stock of their language, training, and behaviour to minimize the possibilities for re-traumatization [81]. Providers are also encouraged to support women's choices based on their own unique circumstances and accept the validity of their concerns [83].

#### Facilitating communication and interaction among peers

Building connections with peers is an important source of support for women in general [68,74,81,83] and has been shown to be particularly important for WLWH [85,94]. Facilitating the development of a network of peers is an integral part of women-specific HIV/AIDS services which may not only serve as a source of support for women but may also help facilitate their access to information. Peers are women who are themselves living with HIV and may share similar life experiences. In-person peer-support groups are one way in which HIV-positive women can share, listen to and support one another [79]. Online peer networking groups are also common. An example of the latter is ViVA, an online community of WLWH in British Columbia, Canada, who share freely with each other about their personal experience, questions and opinions related to HIV/AIDS through a confidential listserv.

#### Involving women in the planning, delivery and evaluation of services

Many HIV-positive people want to participate meaningfully in decisions about the HIV/AIDS services that impact their lives. This was recognized at the international level through the principle of GIPA (Greater Involvement of People Living With HIV/AIDS) [95,96]. This point is particularly salient for WLWH, given their inadequate representation in interventions. Therefore, involving HIV-positive women in the planning, delivery and evaluation of services is an important element to women-specific HIV/AIDS care [81,83]. Policies that employ WLWH are key [78]. Policies that involve them in the planning and decision-making processes through, for example, representation on boards or steering committees [81,84] are also important [67]. Key informant surveys, exit interviews and follow-up evaluations also help ensure that women's perspectives are captured [81]. Applying this principle in practice may require the provision of childcare, transportation, honoraria, mentoring for skill-building or other services to support women's full and equal participation [81]. In addition, encouraging women's involvement also involves actually using their expert knowledge to bring about real change (Margarite Sanchez, Personal Communication, February 2012).

#### Providing self-determination opportunities

Providing self-determination opportunities that aim to help women transform gender norms and achieve equity in outcomes is important for WLWH to contain the epidemic and reduce its impact [8,81]. “[I]mproving access to information, skills, services and technologies” is essential to this approach [8]. Programmes that seek to help women empower themselves recognize the asymmetrical power dynamics between service providers and clients [81]. They seek to promote collaborative work environments that acknowledge women's agency and encourage their equal participation in decision-making about their care [8,67,81,83,84,87]. This is practiced at the Oak Tree Clinic in Vancouver, Canada, where patients are offered a range of services to either select or decline and physicians “stand by them whatever their decision is” [84] (pp. 1408). Women-specific HIV/AIDS services may also have an empowering effect by emphasizing self-worth [64] or by using peer navigators (health advocates who are themselves WLWH) to assist women in building knowledge and skills to navigate the system and be vocal advocates in their own care [81]. They may also address women's oppression at a systemic level by advocating for women's rights [81,87].

### Elements that account for women's unique patterns or preferences in maintaining health and seeking care

#### Providing tailored programming for women

Women-specific approaches to care often include services that are more relevant to women than those provided in mixed-gender settings [62,87]. The overall style of such programming is more supportive, nurturing and cooperative [62,64,68,84]. Programmes often focus on the multiple roles of women, self-worth, emotional safety, physical and sexual abuse, life skills training and strengths identification [64,68,79,89]. Other examples of such programming include women-only support groups, education on women's health topics and female condoms or microbicides along with negotiation skills training to avoid unsafe sex [8,67,76]. Other services typically associated with women's needs include childcare, housing assistance, employment counselling, transportation assistance, family counselling, mental health services and the full spectrum of women's sexual and reproductive health services, including access to safe abortion services [62,66,74,77,79,86]. Promoting women's health also involves efforts to promote healthy lifestyles and screening practices to enable women “to increase control over, and to improve, their health” (Ottawa Charter for Health Promotion).

#### Facilitating meaningful access to care through the provision of social and supportive services

Women-specific HIV/AIDS services understand and respond to the realities of women's lives by providing social and supportive services that facilitate women's access to care. For women with competing care-giving responsibilities, this involves allowing them to be accompanied by their children, providing on-site childcare or offering childcare subsidies [64,68,79]. For low-income women, the provision of travel and transportation support is an important facilitator to care [64,79,83]. Having flexible hours of operation that are “client-driven [and] round the clock” [83,97] (pp. 194) is also key. Other essential practices include being culturally sensitive and offering translation services [78,81,83], ensuring physical accessibility [83] and allowing self-referral to programmes [83]. Providing financially accessible alternative and complementary services is also key [81].

#### Facilitating access to women-specific and culturally sensitive information

In many countries, gender norms often restrict women's access to sexual health information [9,98]. Also, many available HIV resources have been designed for men, resulting in little support catering for women's needs [26,29]. Of the appropriate resources available, there is a lack of culturally- and linguistically appropriate information [30,99]. Limited HIV knowledge can greatly influence women's acquisition and management of the disease [9,98]. Thus, facilitating access to information that is women-specific and culturally sensitive is an important component of women-specific care [8,9,70,78,81]. This requires understanding women's unique learning styles. Women often acquire information through peers, exchanging stories and remembering personal testimonials [81]. Their uptake of information is also shaped by their literacy level, language training and their culture [81]. In response, women-specific services often entail the provision of information in accessible formats, peer-driven education, lunch seminars or women's resource centres [81].

#### Considering family as the unit of intervention

The social reality of HIV-positive women's lives is diverse. Some women are a part of a family unit and make decisions about their health in the context of their family life [81]. In many cases, women's decision-making about accessing and using services and treatments is affected by care-giving responsibilities [36,37] and male partners [8], owing to a power imbalance in gender relations. In other cases, women are either not part of a family unit at all or lack the support mechanisms from family that may be needed to cope with treatment-related issues [26]. Given this diversity, a women-centred approach to care is flexible and takes the different familial contexts of women's lives into consideration.

This type of care respects the role that family may play in a woman's life [62,68,81] and may encompass children, partners and other kin [70,80,84]. This concept of “family-friendly care” is practiced at Vancouver's Oak Tree Clinic, which provides health care to patients’ family members (regardless of their blood relation and HIV status) and a supervised playroom for children [84]. Other examples of this approach to service delivery include HIV pre- and post-test counselling for partners, pregnancy planning with serodiscordant couples and family therapy to help families discuss beliefs about HIV/AIDS [8,80].

In contrast, this type of care may also recognize the harmful gender relations that may exist in families which adversely affect women's access to care and, thus, with a woman's assent, may involve efforts to more fully engage male partners to target these gender norms. For women without family as a social support, efforts to connect women with support mechanisms in their community are also key.

### Philosophies or approaches to delivering women-specific HIV/AIDS services

#### Providing multidisciplinary integration and coordination of a comprehensive array of services

The intersectionality of women's health and social factors means that HIV-positive women face multiple challenges to having their needs met [60]. Managing their illness requires rigorous adherence to combination drug therapies and coordination of multiple specialists which may include primary care providers, psychiatrists, HIV specialists, hepatitis C specialists, social workers, outreach workers, pharmacists, ophthalmologists, gynaecologists, fertility specialists, paediatricians and many others [63,71,90]. Acquiring stable housing, employment, nutritious food, reliable transportation, disability benefits, financial security, child care and other supportive services may also be critical to maintaining health [63,71,84]. Multidisciplinary integration and coordination of an array of services has been promoted as a means for managing the complexities of HIV/AIDS for women [63,64,68,69,72–75,77,80,81,83,84,91]. Achieving this principle in practice often involves one of two models. The first model of service delivery is known as “one-stop shopping” [63,74,81,84,91], in which several services by multiple specialists are offered on-site in one location. The second approach involves an integrated network of services that work in partnership to connect and refer women to the appropriate provider [74], such as case management [71,77,79,80]. This provides a unique opportunity for the coordination of various services where service fragmentation is common [63,71]. Both approaches require collaborative planning and delivery of care by an interdisciplinary team of providers [63,81,83].

#### Meeting women “where they are”

A SDoWH framework highlights how WLWH have multiple overlapping needs and are at various stages in their lives and in their experiences of HIV. A women-specific approach to HIV service delivery acknowledges this by meeting “women where they are” [82,85]. This involves supporting each woman by adequately meeting her individual health and social needs and by being all of whom she wants to be without passing judgment [85]. It also involves a commitment to flexibility, adjusting care for different needs and stages in a woman's life [73,83,87]. In practice, this may mean not chastising women for a missed appointment but simply re-scheduling it. It may also mean delivering care directly to a woman's home if she has to care for a disabled child or parent at home. It also includes services that reflect the realities of WLWH, such as those with flexible hours of operation for women with daytime commitments or those with childcare provisions for women with young children and no access to childcare.

### Methods that inform a women-specific approach to HIV/AIDS care

#### Providing gender-, culture- and HIV-sensitive training to health and social care providers

Many WLWH have reported negative experiences with service providers due to intersectional discrimination against being a woman, HIV-positive, Aboriginal, Black or being from an HIV-endemic country, being lesbian, bisexual or transgender [29–32]. Women have also reported a lack of knowledge among physicians regarding the impact of antiretroviral medications on women's bodies such as changes in menstrual cycles, weight and fat distribution [26]. In response, women-specific services often include gender-, culture- and HIV-sensitive training to help providers improve their understanding of WLWH and of different cultures, health practices and beliefs [70,81,84]. This begins with providers taking stock of their own assumptions and incorporating into practice only those values that support women-centred health [81]. It also involves continuing education on issues specific to women, culture and HIV. Facilitating this involves the provision of regular information updates, awareness workshops and comprehensive sensitivity training presented by diverse WLWH [70,81]. According to a SDoWH framework, attention to these intersecting social identities is necessary to understand their combined influence on health and to develop more effective health services [56,57,60].

#### Conducting gendered HIV/AIDS research

Historically, HIV-positive women have been inadequately represented in HIV/AIDS research [6,100]. Results from studies involving mainly men are not necessarily generalizable to women [101] and there is a critical need to address issues of gender in research to effectively respond to HIV in women in practice [6]. The importance of applying a gendered lens to interventions addressing the health of WLWH has been highlighted in the SDoWH literature [56,57,60]. In response, some women-specific care programmes have begun conducting their own gendered research initiatives [81]. This approach involves the participation of patients in onsite or offsite research studies. It also requires a breakdown by gender in all data and a consideration of gendered issues in all phases of the research process [101]. Use of multi-methods is also valuable as “women's voices are an important part of evidence” [81]. In this way, care programmes themselves can identify and address issues and gaps in service delivery [81].

### Conceptual framework of holistic service delivery for WLWH

The previous sections provided a synthesis of the defining characteristics and other major elements of holistic service delivery for WLWH. After reviewing the literature, it emerged that recognizing and responding to women's unique health and social care needs is more at the core of HIV programming for women than being women-specific (or separate from men) *per se*. Thus, we propose the use of the term “women-centred HIV/AIDS services” in our conceptual framework and in future empirical research in this area since it better reflects the literature reviewed and our SDoWH theoretical approach. We now propose the following framework (adapted from Vancouver/Richmond Health Board) to illustrate this concept, positioning it within its broader context of the lives of HIV-positive women.

**Figure 2:**
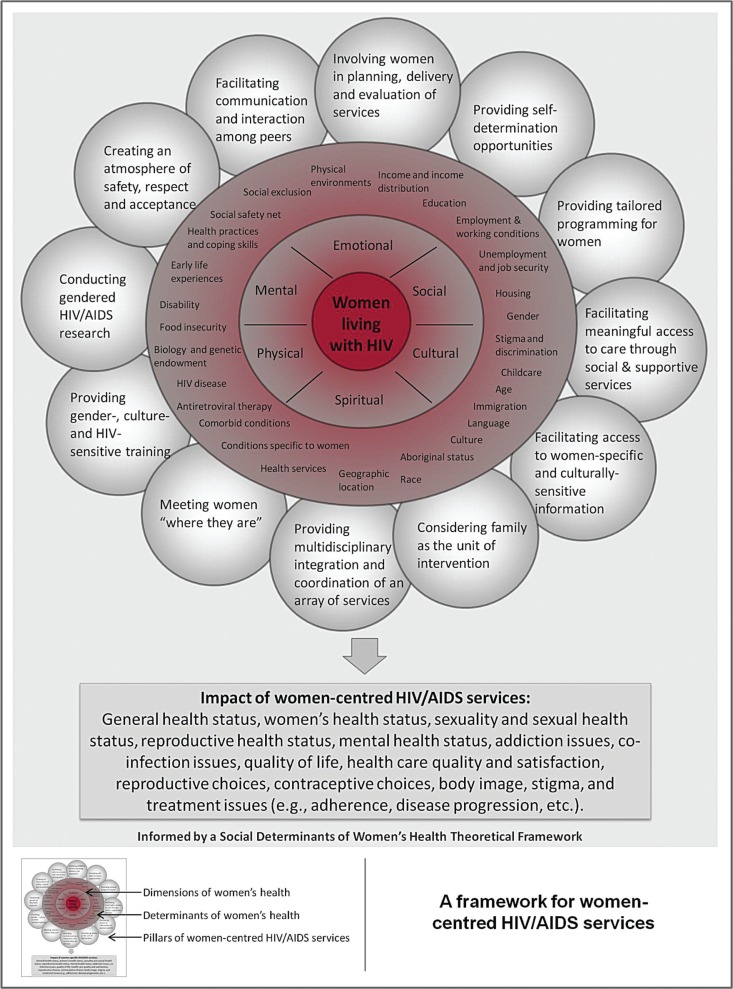
A conceptual framework of the concept of women-centred HIV/AIDS services, placed within the context of the lives of women living with HIV (WLWH). Adapted from Vancouver/Richmond Health Board [81]. WLWH are at the centre of the framework and are encircled, first, by six dimensions of women's health, and, second, by 28 determinants of women's health. Around this are 12 components, or pillars, of women-centred HIV/AIDS services. The box below summarizes the potential impact of these services on HIV-positive women's health outcomes. An SDoWH theoretical perspective overlays the entire framework as outlined in the final box enclosing the diagram.

In Figure 2, WLWH have been placed in the middle of the framework to indicate the centrality of women to women-centred HIV/AIDS services. Women are then encircled by six dimensions of women's health: emotional, mental, social, cultural, spiritual and physical wellbeing [92]. This circle, designed as a wheel with spokes, represents women's overall well-being and illustrates the importance of maintaining balance between the six different aspects of one's health.

Women's health is determined by the context of their lives as well as by biology. As such, the next concentric circle shows 28 determinants of women's health. Of these, 14 were described by Raphael (Aboriginal status, gender, disability, housing, early life experiences, income and income distribution, education, race, employment and working conditions, social exclusion, food insecurity, social safety net, health services and unemployment and job security) [102], three were supported by Health Canada (biology and genetic endowment, physical environments (i.e., neighbourhood safety) and personal health practices and coping skills) [103] and seven were added from the literature on SDoWH [56,57,60] to better reflect the realities of WLWH (age, immigration, geographic location, childcare, culture, language and stigma and discrimination). This circle also includes four other elements relevant to the physical dimension of HIV-positive women's health, including HIV disease, antiretroviral therapy, comorbid conditions (e.g. Hepatitis C), and conditions specific to women (e.g. gynaecologic diseases).

In the outermost layer, the 12 components, or pillars, of women-centred HIV/AIDS services that we identified in this review are displayed. As shown, the components overlap and are not mutually exclusive; rather, in practice, they may operate either alone or in combination with other elements simultaneously depending on the context. The box at the bottom of the diagram provides a synthesis of the documented and hypothesized impacts of women-centred HIV/AIDS services on various health, social and treatment outcomes of WLWH.

The entire framework is informed by an SDoWH theoretical perspective as outlined by the final box enclosing the diagram. Grounded in social justice and human rights [104], this paradigm highlights how, with the exception of the work of peers, self-determination and GIPA, there have been few attempts in the literature to frame this issue beyond that of individualized interventions that are focused on the private sphere of one's life (e.g. decision-making, self-worth, family). This places emphasis on changing or controlling individual behaviours and deflects attention away from the broader socio-structural forces (e.g. poverty, patriarchy and other forms of structural violence) that systematically deny women's rights and well-being [104]. Therefore, the inclusion of this final box is meant to highlight the importance of understanding and addressing these social, economic and political structures to the promotion of women's health.

## Limitations

The concept of women-specific HIV/AIDS services was shaped through a review of the perspectives of experts from many different disciplines. Thus, the data were fragmented across many subject areas and the information outlined by some theorists may not be perfectly relevant to the context of HIV/AIDS. In addition, the examples of how each of the 12 pillars to HIV care for women is applied in practice were not exhaustive and efforts should be made to expand on the breadth of the components. Furthermore, it was difficult to identify clear-cut components and, thus, there is overlap between some of the 12 pillars. Finally, most articles included in this review originated from the US, Canada, and Europe. Since models of HIV health care coverage and delivery differ worldwide, the recommendations in this article may not be generalizable beyond these settings.

Despite these limitations, there are also strengths stemming from the information gathered. The inclusion of articles from multiple fields helped to create a rich and textured understanding of women-specific HIV/AIDS services that holistically reflects women's lives. Also, while the framework is simplified, it nonetheless provides an overall picture of the various factors that make up a women-specific approach to HIV care. Thus, it is hoped that this framework can help guide future HIV care and research aimed at developing, measuring and evaluating women-specific HIV/AIDS services.

## Conclusions: implications for practice and research

The evolving demographics of the epidemic and the underlying gender dynamics necessitate a tailored approach to service delivery worldwide that is responsive to the unique needs of WLWH and is guided by prevailing regional and local conditions. As demonstrated in this review, the concept of women-specific HIV/AIDS services is a complex and multidimensional one that has been shaped by diverse theoretical perspectives. The framework outlined in this article provides a useful tool that can assist health planners and providers to improve women-centred approaches to HIV care so that the system better meets the needs of WLWH. Further research is needed to better understand this emerging concept and ultimately assess the effectiveness of women-specific HIV/AIDS services in achieving optimal health outcomes for WLWH. This work will be undertaken in the next phase of CHIWOS and will have important implications for evidence-based holistic health services for HIV-positive women in Canada and worldwide.

## The CHIWOS Research Team

Principal Investigators: Mona R. Loutfy (*Women's College Research Institute*), Alexandra de Pokomandy (*McGill University Health Centre*), Angela Kaida (*Simon Fraser University*) and Bob Hogg (*British Columbia Centre for Excellence in HIV/AIDS and Simon Fraser University*). Community-Based Research Consultant: Shari Margolese (*Women's College Research Institute*). Research Coordinators: Kira Abelsohn (*Women's College Research Institute*), Allison J. Carter (*British Columbia Centre for Excellence in HIV/AIDS and Simon Fraser University*), Nadia O'Brien (*McGill University Health Centre*). Research Associates: Dada Bakombo, Cindy Lou Brisebois, Janice Dayle, Haoua Inoua, Gladys Kwaramba, Kecia Larkin, Brigitte Ménard, Valerie Nicholson, Stephanie Rawson, Stephanie Smith. National Core Research Team Members: Kira Abelsohn (*Women's College Research Institute*), Aranka Anema (*British Columbia Centre For Excellence in HIV/AIDS*), Dada Bakombo, Cindy Lou Brisebois, Allison J. Carter (*British Columbia Centre for Excellence in HIV/AIDS and Simon Fraser University*), Angela Cescon (*British Columbia Centre for Excellence in HIV/AIDS*), Janice Dayle, Alexandra de Pokomandy (*McGill University Health Centre*), Bob Hogg (*British Columbia Centre for Excellence in HIV/AIDS and Simon Fraser University*), Saara Greene (*McMaster University*), Haoua Inoua, Angela Kaida (*Simon Fraser University*), Gladys Kwaramba, Kecia Larkin, Johanna Lewis (*Women's College Research Institute*), Mona R. Loutfy (*Women's College Research Institute*), Shari Margolese (*Women's College Research Institute*), Brigitte Ménard, Valerie Nicholson, Nadia O'Brien (*McGill University Health Centre*), Ali Palmer (*British Columbia Centre for Excellence in HIV/AIDS*), Stephanie Rawson, Stephanie Smith, Wangari Tharao (*Women's Health in Women's Hands*), Ann Burchell (*Ontario HIV Treatment Network*). Provincial Core Research Team Members: BC: Allison J. Carter (*British Columbia Centre for Excellence in HIV/AIDS and Simon Fraser University*), Angela Cescon (*British Columbia Centre for Excellence in HIV/AIDS*), Bob Hogg (*British Columbia Centre for Excellence in HIV/AIDS and Simon Fraser University*), Angela Kaida (*Simon Fraser University*), Kecia Larkin, Valerie Nicholson, Ali Palmer (*British Columbia Centre for Excellence in HIV/AIDS*), and Stephanie Rawson. ON: Kira Abelsohn (*Women's College Research Institute*), Anita Benoit (*Women's College Research Institute*), Cindy Brisebois, Ann Burchell (*Ontario HIV Treatment Network*), Saara Greene (*McMaster University*), Haoua Inoua, Logan Kennedy (*Women's College Research Institute*), Gladys Kwaramba, Johanna Lewis (*Women's College Research Institute*), Mona R. Loutfy (*Women's College Research Institute*), Shari Margolese (*Women's College Research Institute*), Samantha Robinson (*Ontario HIV Treatment Network*), Stephanie Smith, Wangari Tharao (*Women's Health in Women's Hands*). QC: Dada Bakombo, Mélina Bernier (*Université du Québec à Montréal*), Janice Dayle, Alexandra de Pokomandy (*McGill University Health Centre*), Lyne Massie, Brigitte Ménard, Nadia O'Brien (*McGill University Health Centre*), Joanne Otis (*Université du Québec à Montréal*). National Steering Committee Members: Kira Abelsohn (*Women's College Research Institute*), Pat Allard (*Canadian HIV/AIDS Legal Network*), Aranka Anema (*British Columbia Centre For Excellence in HIV/AIDS*), Dada Bakombo, Denise Becker (*Positive Living Society of British Columbia*), Mélina Bernier (*Université du Québec à Montréal*), Cindy Lou Brisebois, Jason Brophy (*Children's Hospital of Eastern Ontario*), Ann Burchell (*Ontario HIV Treatment Network*), Allison J. Carter (*British Columbia Centre for Excellence in HIV/AIDS and Simon Fraser University*), Angela Cescon (*British Columbia Centre for Excellence in HIV/AIDS*), Janice Dayle, Alexandra de Pokomandy (*McGill University Health Centre*), Jacqueline Gahagan (*Dalhousie University*), Saara Greene (*McMaster University*), Bob Hogg (*British Columbia Centre for Excellence in HIV/AIDS and Simon Fraser University*), Terry Howard (*Positive Living Society of British Columbia*), Haoua Inoua, Evin Jones (*Pacific AIDS Network*), Angela Kaida (*Simon Fraser University*), Alexandria Keating (*ViVA and Southern Gulf Islands AIDS Society (SGIAS)*), Marina Klein (*McGill University Health Centre*), Gladys Kwaramba, Kecia Larkin, Rebecca Lee (*CIHR Canadian HIV Trials Network*), Johanna Lewis (*Women's College Research Institute*), Mona Loutfy (*Women's College Research Institute*), Shari Margolese (*Women's College Research Institute*), Renee Masching (*Canadian Aboriginal AIDS Network*), Melissa Medjuck (*Positive Women's Network*), Brigitte Ménard, Deb Money (*Women's Health Research Institute*), Valerie Nicholson, Nadia O'Brien (*McGill University Health Centre*), Susanna Ogunnaike-Cooke (*Public Health Agency of Canada*), Joanne Otis (*Université du Québec à Montréal*), Sara O'Shaughnessy (*Pacific AIDS Network*), Ali Palmer (*British Columbia Centre for Excellence in HIV/AIDS*), Doris Peltier (*Canadian Aboriginal AIDS Network*), Neora Pick, Janet Raboud (*Ontario HIV Treatment Network*), Stephanie Rawson, Samantha Robinson (*Ontario HIV Treatment Network*), Sean Rourke (*Ontario HIV Treatment Network*), Margarite Sanchez (*ViVA and Southern Gulf Islands AIDS Society (SGIAS)*), Jacquie Sas (*CIHR Canadian HIV Trials Network*), Stephanie Smith, Marcie Summers (*Positive Women's Network*), Wangari Tharao (*Women's Health in Women's Hands*), Sharon Walmsley (*Toronto General Research Institute*), Jessica Yee (*Native Youth Sexual Health Network*). Sampling, Recruitment and Data Management and Analyst Team Members: Ann Burchell (*Ontario HIV Treatment Network*), Allison J. Carter (*British Columbia Centre for Excellence in HIV/AIDS and Simon Fraser University*), Guillaume Colley (*British Columbia Centre for Excellence in HIV/AIDS*), Alexandra de Pokomandy (*McGill University Health Centre*), Nada Gataric (*British Columbia Centre for Excellence in HIV/AIDS*), Angela Kaida (*Simon Fraser University*), Johanna Lewis (*Women's College Research Institute*), Dr. Mona R. Loutfy (*Women's College Research Institute*), Nadia O'Brien (*McGill University Health Centre*), Janet Raboud (*Ontario HIV Treatment Network*), Jayson Shurgold, Wangari Tharao (*Women's Health in Women's Hands*) and Wendy Zhang (*British Columbia Centre for Excellence in HIV/AIDS*).
